# Induction of Drought Tolerance in Cucumber Plants by a Consortium of Three Plant Growth-Promoting Rhizobacterium Strains

**DOI:** 10.1371/journal.pone.0052565

**Published:** 2012-12-28

**Authors:** Chun-Juan Wang, Wei Yang, Chao Wang, Chun Gu, Dong-Dong Niu, Hong-Xia Liu, Yun-Peng Wang, Jian-Hua Guo

**Affiliations:** 1 Department of Plant Pathology, College of Plant Protection, Nanjing Agricultural University, and Key Laboratory of Integrated Management of Crop Diseases and Pests, Nanjing Agricultural University, Ministry of Education, Nanjing, China; 2 Anfeng Biogenic Pesticide Engineering Center of Jiangsu Province, Taicang, China; 3 Jiangsu Key Laboratory for Eco-Agricultural Biotechnology around Hongze Lake, School of Life Science, Huaiyin Normal University, Huai'an, China; University of Wisconsin-Milwaukee, United States of America

## Abstract

Our previous work showed that a consortium of three plant growth-promoting rhizobacterium (PGPR) strains (*Bacillus cereus* AR156, *Bacillus subtilis* SM21, and *Serratia* sp. XY21), termed as BBS for short, was a promising biocontrol agent. The present study investigated its effect on drought tolerance in cucumber plants. After withholding watering for 13 days, BBS-treated cucumber plants had much darker green leaves and substantially lighter wilt symptoms than control plants. Compared to the control, the BBS treatment decreased the leaf monodehydroascorbate (MDA) content and relative electrical conductivity by 40% and 15%, respectively; increased the leaf proline content and the root recovery intension by 3.45-fold and 50%, respectively; and also maintained the leaf chlorophyll content in cucumber plants under drought stress. Besides, in relation to the control, the BBS treatment significantly enhanced the superoxide dismutase (SOD) activity and mitigated the drought-triggered down-regulation of the expression of the genes *cAPX*, *rbcL*, and *rbcS* encoding cytosolic ascorbate peroxidase, and ribulose-1,5-bisphosphate carboxy/oxygenase (Rubisco) large and small subunits, respectively, in cucumber leaves. However, 1-aminocyclopropane-1-carboxylate (ACC) deaminase activity was undetected in none of the culture solutions of three BBS constituent strains. These results indicated that BBS conferred induced systemic tolerance to drought stress in cucumber plants, by protecting plant cells, maintaining photosynthetic efficiency and root vigor and increasing some of antioxidase activities, without involving the action of ACC deaminase to lower plant ethylene levels.

## Introduction

Due to their sessile nature, plants are constantly faced with abiotic and biotic stresses during their whole life time. Abiotic stresses are various adverse environmental factors, including drought, high salt, heavy metals, cold or heat shock, and ozone. Resulting in dehydration and osmotic stress, drought has caused a dramatic reduction in crop production globally [1]–[4]. On the other hand, a range of abiotic and biotic elicitors can confer tolerance to drought stress in plants. The former includes alginate-derived oligosaccharides [5], ketoconazole [6], CO_2_ laser [7], 2-aminoethanol [8], abscisic acid (ABA) [9], and brassinosteroids [10], [11], while the latter comprises rhizobia [12], [13], mycorrhizal fungi [14]–[18], endophytic fungus [19], [20] as well as additional types of beneficial microorganisms [21]–[24]. These elicitors can reduce the content of monodehydroascorbate (MDA); prevent the accumulation of reactive oxygen species (ROS); increase activities of antioxidant enzymes [5], [7]–[11], [23], [24]; and maintain fresh and dry weights, grain yield, and relative water content in a variety of plants in response to drought stress [10], [18], [22]–[24]. Compared to abiotic elicitors, the biotic has more superiority. For example, beneficial microorganisms can stably colonize the rhizosphere of various plant species, and promote plant growth by improving soil structure and moisture retention as well as by enhancing plant mineral-nutrition absorption, etc [15]. Furthermore, plant growth-promoting rhizobacterium (PGPR) such as *Bacillus cereus* AR156 can enhance disease resistance [25].

PGPR induces physical and chemical changes in plants, resulting in enhanced plant tolerance to abiotic stresses termed as induced systemic tolerance (IST) [26]. It is known that PGPR confers IST to drought stress in plants by a variety of mechanisms. For instance, the PGPR strain *Paenibacillus polymyxa* has been demonstrated to enhance the drought tolerance of *Arabidopsis thaliana* by stimulating the transcription of a drought-response gene, EARLY RESPONSIVE TO DEHYDRATION 15 (ERD15), and of an ABA-responsive gene, *RAB18* [21]. In addition, it has been well established that PGPR strains that contain 1-aminocyclopropane-1-carboxylate (ACC) deaminase confer IST to drought stress in a number of plants via the action of ACC deaminase to lower plant ethylene levels. For example, the ACC deaminase-containing PGPR strain *Achromobacter piechaudii* ARV8 has been demonstrated to significantly increase the fresh and dry weights of both drought-treated tomato and pepper seedlings, and reduce ethylene production in tomato seedlings exposed to transient water deficit stress [22]. Furthermore, *Variovorax paradoxus* 5C-2, another PGPR strain producing ACC deaminase, has been shown to reduce abscission of the mature leaves of *Cytisus*×*praecox* and late season senescence in *Aquilegia*×*hybrida* experiencing drought stress by lowering ethylene emission in these plants [27].

In addition to single strains of PGPR, its combination with either mycorrhizal fungi or *Rhizobium* also has also been demonstrated to elicit plant drought tolerance. For instance, co-inoculation of the common bean (*Phaseolus vulgaris* L.) with *Rhizobium tropici* (CIAT 899) and the two *Paenibacillus* strains *Paenibacillus polymyxa* (DSM 36) and *Paenibacillus polymyxa* Loutit (L) more effectively alleviated the deleterious effects of drought stress on plant growth, nitrogen content, and nodulation than inoculation with *R. tropici* (CIAT 899) alone [28]. Moreover, co-inoculation of lettuce with the PGPR strain *Pseudomonas mendocina* Palleroni and an arbuscular mycorrhizal (AM) fungus (either *Glomus intraradices* or *Glomus mosseae*) significantly enhanced the root phosphatase activity; and the proline accumulation and the activities of nitrate reductase, peroxidase (POD), and catalase (CAT) in the leaves under moderate and severe drought stress [29].

Usually grown in the greenhouse, cucumber (*Cucumis sativus* L.) plants need much water during their life time, so drought stress is a limiting factor for their growth and development. While a variety of abiotic elicitors, including brassinosteroids [10], [11], silicon [30], external trehalose [31], salicylic acid, oxalic acid, and proline [32], were found to induce cucumber drought tolerance, few biotic ones have been shown to possess this ability. Referred to as BBS for short, a consortium of three PGPR strains (*Bacillus cereus* AR156, *Bacillus subtilis* SM21, and *Serratia* sp. XY21) was demonstrated to be a promising biocontrol agent in our earlier research and brand named as ‘Shu Dekang’ [33], since it significantly inhibited a number of plant diseases, including the leaf speck disease caused by *Pseudomonas syringae* pv. *tomato* in tomato plants [34], the banana wilt caused by *Fusarium oxysporum* f. sp. *cubense* [35], the blight caused by *Phytophthora capsici* Leon. in hot pepper plants (unpublished data), and the root-knot disease caused by *Meloidogyne incognita* in bitter melon [36] and cucumber (*Cucumis sativus* L.) plants [37]. Aiming at assessing its potential for inducing cucumber drought tolerance, here, we examined the effects of BBS on a range of physiological indicators of drought tolerance, the activities of antioxidant enzymes, and expression profiles of the genes encoding cytosolic ascorbate peroxidase (*cAPX*) and ribulose-1,5-bisphosphate carboxy/oxygenase (Rubisco) large and small subunits (*rbcL* and *rbcS*) in cucumber plants experiencing drought stress. Results suggested that BBS conferred IST to drought in cucumber plants, without involving the action of ACC deaminase.

## Results

### BBS inducing drought tolerance in cucumber plants

**Figure 1:**
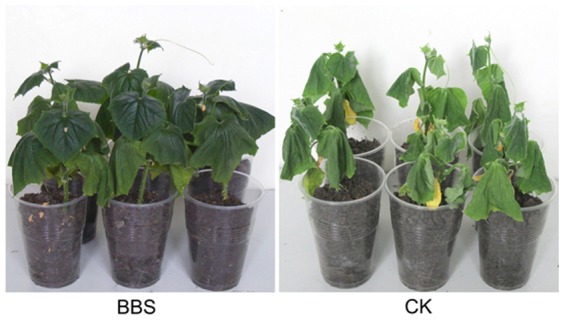
BBS inducing drought tolerance in cucumber plants. The cell suspension of BBS and sterile water were poured on the soil around cucumber roots in the BBS treatment (BBS) and the control (CK), respectively, and then watering was withheld for 13 days.

After withholding watering for 13 days, the leaves of BBS-treated cucumber plants displayed much darker green and lighter wilt symptoms (i.e. still being able to rehydrate at night or in an early morning) than those of control plants, which were unable to rehydrate at night or in an early morning (Figure 1).

**Figure 2:**
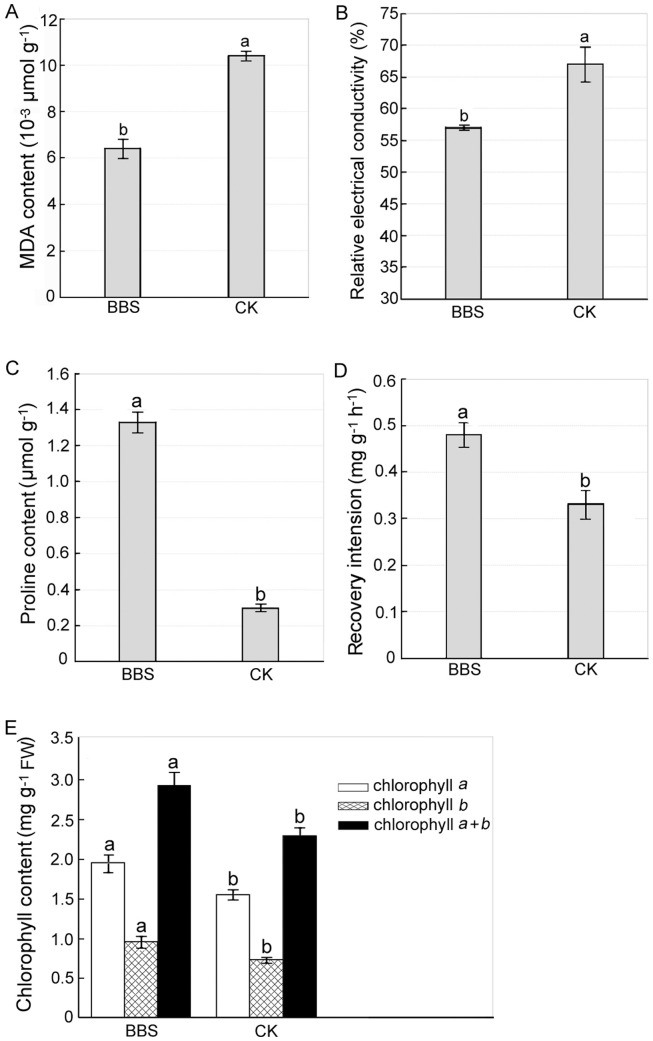
Effects of BBS on physiological indicators of cucumber drought tolerance. The cell suspension of BBS and sterile water were poured on the soil around cucumber roots in the BBS treatment (BBS) and the control (CK), respectively, and then watering was withheld for 13 days. The leaf MDA content (A), the leaf relative electrical conductivity (B), and the leaf proline content (C), the root recovery intension (D), and the leaf chlorophyll content (E) of cucumber plants under drought stress were determined at 13 dpi. Each treatment was replicated three times; data are presented as means of three replicates ± SD, and error bars represent SD for three replicates. Means with different letters have significant differences (p<0.05; LSD test).

It is known that the imposition of drought on plants leads to enhanced membrane peroxidation in leaf tissues, thus increasing the leaf MDA content and relative electrical conductivity. Therefore, we determined the leaf MDA content and relative electrical conductivity in cucumber plants. Under drought stress the leaf MDA content and relative electrical conductivity in BBS-treated cucumber plants were 3.2×10^−3^ µmol/g and 57.0%, respectively, which decreased by 38.5% (Figure 2A) and 14.9% (Figure 2B), respectively, compared with their counterparts in control plants.

Increasing leaf proline content is crucial for maintaining the osmotic potential of leaf tissues, which plays an important role in protecting them from over-dehydration under drought stress. The application of BBS resulted in a remarkable 3.4-fold increase in the leaf proline content in cucumber plants compared with the control (1.33 µmol/g vs. 0.30 µmol/g) (Figure 2C). Furthermore, as a reliable and sensitive indicator of plant drought tolerance, the root recovery intension significantly increased by 45.5% in BBS-treated cucumber plants (Figure 2D) in relation to that in control plants.

### BBS maintaining leaf chlorophyll content and transcriptional levels of *rbcS* and *rbcL*

In order to examine the impact of BBS on the photosynthetic efficiency of cucumber plants exposed to water deficit stress, we first determined the leaf chlorophyll content in cucumber plants. The contents of leaf chlorophylls *a*, *b*, and *a*+*b* in BBS-treated cucumber plants increased by 25.9%, 31.5%, and 27.4%, respectively, in comparison with their counterparts in control plants (Figure 2E), which suggested that BBS maintained chlorophyll contents in cucumber leaves under drought stress. This was consistent with the above observation that BBS-treated cucumber plants had much darker green leaves than control plants (Figure 1).

**Figure 3:**
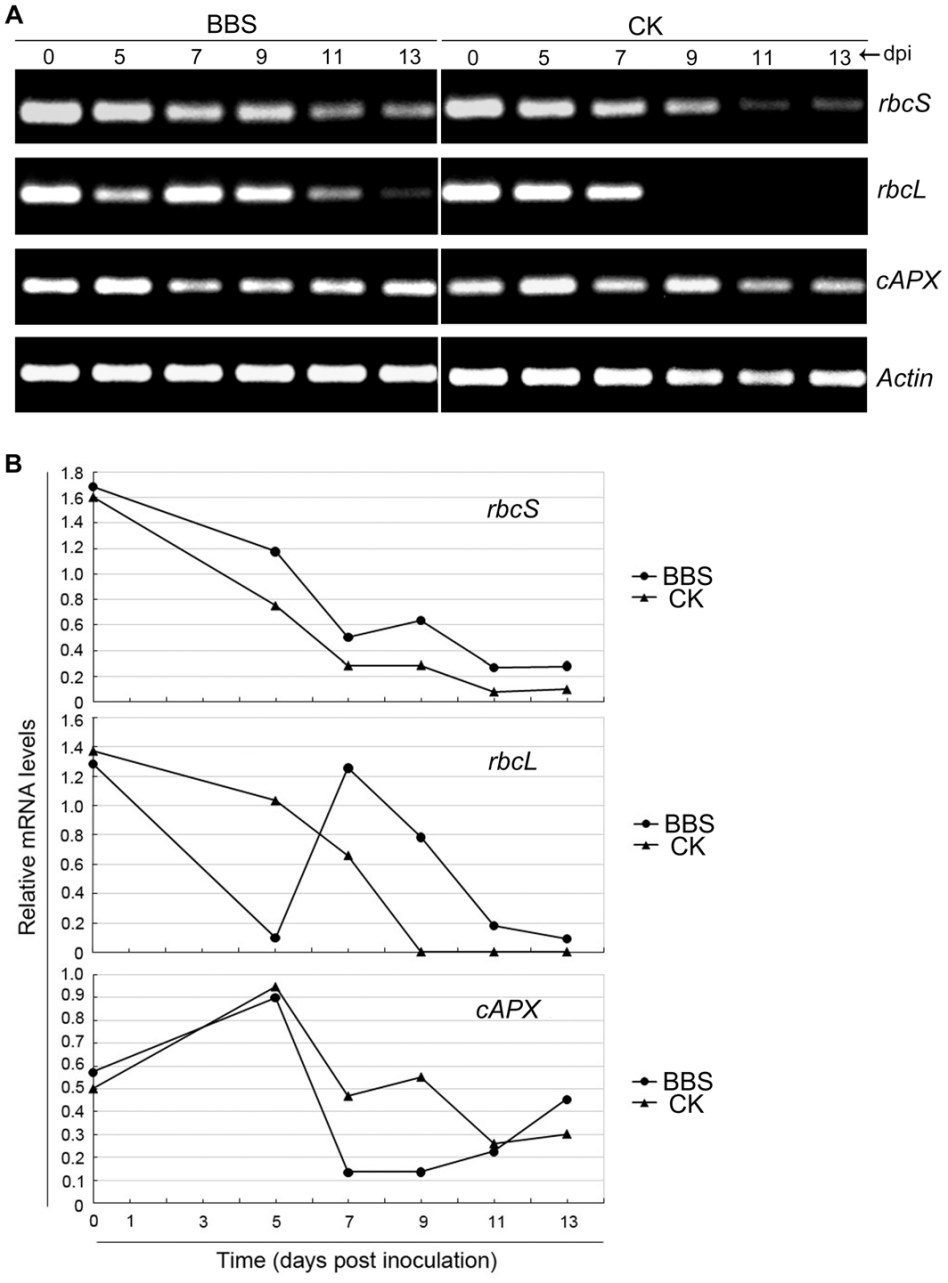
Effects of BBS on the expression of the *rbcS*, *rbcL*, and *cAPX* genes. The cell suspension of BBS and sterile water were poured on the soil around cucumber roots in the BBS treatment (BBS) and the control (CK), respectively, and then watering was withheld for 13 days. A, The expression of the genes *rbcS*, *rbcL* and *cAPX* was detected at 0, 5, 7, 9, 11, and 13 dpi by RT-PCR. B, Relative mRNA levels of genes based on Quality One software of Bio-Rad. Each treatment was replicated three times. dpi, day post inoculation; *Actin*, internal reference.

It is well known that Rubisco, a bifunctional enzyme located in the chloroplast stoma, catalyzes photosynthetic CO_2_ fixation to form ribulose-1,5-bisphosphate (RuBP) [38]; therefore, the transcriptional levels of the *rbcS* and *rbcL* genes in cucumber leaves may indicate their photosynthetic efficiency. We analyzed the transcriptional patterns of the two genes in cucumber leaves under drought conditions. While the transcriptional level of the *rbcS* gene gradually declined along with extending time of water deprivation in both the BBS and control treatment, it was down-regulated to a smaller extent in the BBS treatment than in the control (Figure 3A, B). Specifically, although the *rbcL* transcription was down-regulated sharply at 5 dpi, it was robust at 7 dpi and still detected at 11 and 13 dpi in the BBS treatment, but undetected since 9 dpi in the control (Figure 3A, B), indicating that the *rbcL* transcription was stronger in the BBS treatment than that in the control over 7–13 dpi. This demonstrated that BBS maintained the transcriptional levels of *rbcS* and *rbcL* genes in cucumber leaves under aggravated drought stress.

### Effects of BBS on SOD, POD, and CAT activities; and on *cAPX* expression

**Figure 4:**
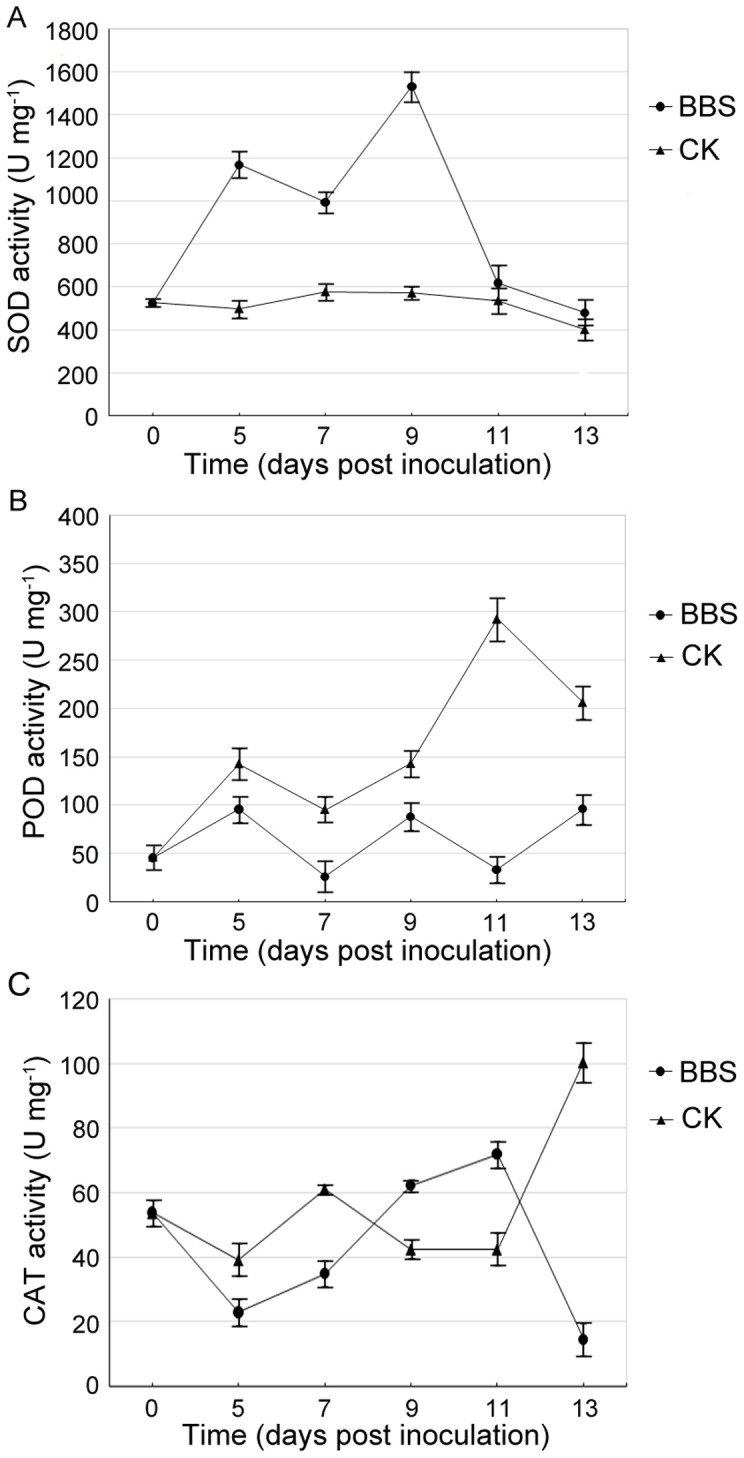
Effects of BBS on SOD, POD, and CAT activities. The cell suspension of BBS and sterile water were poured on the soil around cucumber roots in the BBS treatment (BBS) and the control (CK), respectively, and then watering was withheld for 13 days. The leaf SOD, POD, and CAT activities in cucumber plants under drought stress were determined at 0, 5, 7, 9, 11, and 13 dpi. Each treatment was replicated three times; data are presented as means of three replicates ± SD, and error bars represent SD for three replicates. (p<0.05; LSD test).

It has been well established that drought stress promotes the production of oxygen free radicals in plants, which, however, can be scavenged and detoxified by plant enzymatic- and non-enzymatic systems to protect it from oxidative damage. We investigated the impact of BBS on the corresponding enzymatic system of cucumber plants in response to drought stress by determining the leaf SOD, POD, and CAT activities. As shown in Figure 4A, the application of BBS led to a substantial increase in the leaf SOD activity over 0–9 dpi, which peaked at 9 dpi (1533.17 U/mg) and then sharply fell until 11 dpi, but its counterpart in the control remained low over the entire time course 0–13 dpi. This demonstrated that BBS significantly stimulated the leaf SOD activity in cucumber plants under drought stress.

However, over 0–13 dpi, the leaf POD activity in BBS-treated cucumber plants fluctuated between 26.30 and 96.18 U/mg, while its counterpart in control plants exhibited an overall increasing trend by peaking at 292.94 U/mg at 11 dpi (Figure 4 B). CAT is another enzyme to scavenge H_2_O_2_. The leaf CAT activity in the BBS treatment firstly decreased, then continuously increased over 5–11 dpi, and finally dropped sharply to 14.74 U/mg at 13 dpi, which was higher at 9 and 11 dpi; but lower at 5, 7, and 13 dpi compared to the counterpart in the control (Figure 4C). Thus, overall, the BBS treatment did not enhance the CAT activity compared to the control. Besides, the expression of the gene *cAPX* encoding cytosolic ascorbate peroxidase, another H_2_O_2_ scavenging enzyme, was examined at the same set of time points in the BBS and control treatments. The two treatments were found to be similar in the fashion of the *cAPX* transcription over 0–7 dpi, which was slightly enhanced at 5 dpi and then noticeably lessened at 7 dpi; however, over 9–13 dpi, its level kept increasing and almost recovered to the initial level at 13 dpi in the BBS treatment, but substantially dropped at 11 dpi following an increase at 9 dpi and remained relatively low at 13 dpi in the control (Figure 3A, B). This demonstrated that BBS maintained *cAPX* transcriptional levels in cucumber plants in response to extending time of water deprivation.

### All three BBS constituent strains containing no ACC deaminase

**Figure 5:**
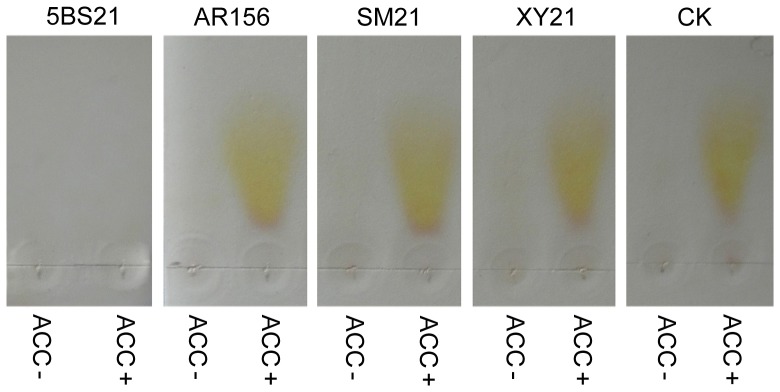
Detection of ACC deaminase activity in three BBS constituent strains and *Burkholderia* sp. 5BS21. After grown in MSA medium with ACC (ACC+) or without ACC (ACC−) for 48 h, bacterial cells of *Burkholderia* sp. 5BS21 (5BS21) and of the three BBS constituent strains *Bacillus cereus* AR156 (AR156), *Bacillus subtilis* SM21 (SM21), *Serratia* sp. XY21 (XY21) were centrifuged at 14000× g for 5 min. Then 5 µL of the supernatant resulting from each culture was spotted on a TLC silica gel plate with 5 µL of MSA medium with ACC (ACC+) and 5 µL of that without ACC (ACC−) as controls. The gel was developed for 2.5 h and then stained with 0.5% ninhydrin for 20 min in an attemperator set at 100°C.

We detected the activity of ACC deaminase in all three BBS constituent strains, *Bacillus cereus* AR156 (AR156), *Bacillus subtilis* SM21 (SM21), and *Serratia* sp. XY21 (XY21), to investigate whether they confer IST to drought stress in cucumber plants by producing ACC deaminase to degrade the ethylene precursor ACC. As a result, the activity of ACC deaminase was undetected in all these strains, but detected in the known ACC deaminase-containing strain *Burkholderia* sp. 5BS21 (5BS21) (Figure 5). Correspondingly, homologous genes of ACC deaminase were undetected in AR156, SM21 and XY21 genome through PCR (data not showed). These implicated that BBS-elicited IST to drought stress in cucumber plants did not involve the action of ACC deaminase to lower ethylene levels in cucumber plants.

## Discussion

In this study BBS revealed its ability to confer IST to drought stress in cucumber plants, which was manifested by that BBS-treated plants had significantly lighter wilt symptoms and much darker green leaves than control plants after withholding watering for 13 days (Figure 1). Our results also implicated a number of mechanisms underlying BBS-elicited IST to drought. The reductions in the leaf MDA content and relative electrical conductivity resulting from the BBS treatment (Figures 2A and 2B) suggest its capability for reducing the extent of the peroxidation of plasma lemma under drought stress to protect the leaf cell membrane from damage. Also, a remarkable increase in the leaf proline content in the BBS treatment (Figure 2C) reflects its effectiveness in stabilizing the osmotic potential in cucumber leaves under drought stress. In addition, the relatively high levels of root recovery intension noted in BBS-treated plants under drought stress (Figure 2D) implicate its role in protecting the roots from the detrimental effects of drought stress. Moreover, significantly higher chlorophyll content (Figure 2E) and smaller extent of down-regulation of *rbcL* and *rbcS* transcription in the leaves of BBS-treated cucumber plants (Figure 3) in relation to control plants suggest its ability to maintain photosynthesis efficiency under water deficit conditions.

To further elucidate the underlying mechanisms of BBS-elicited IST to drought in cucumber plants, in this study we also investigated its effects on the activities of a variety of antioxidases. It is known that drought stress promotes the production of ROS, including superoxide (O_2_^−^), singlet oxygen (^.^O_2_), hydroxyl (OH^−^), and hydrogen peroxide (H_2_O_2_), which exert oxidative stress on plants [39]–[41]. On the other side, plants have evolved both enzymatic and non-enzymatic defense systems for scavenging and detoxifying ROS. In the enzymatic defense system, SOD scavenges O_2_^−^ by transforming it into H_2_O_2_ [42]. Xia et al. [43] reported that elevated H_2_O_2_ levels resulting from enhanced triphosphopyridine nucleotide (NADPH) oxidase activity are involved in the brassinosteroids-induced drought stress tolerance in cucumber plants. In this study the application of BBS increased the activity of SOD in cucumber leaves (Figure 4A), suggesting that BBS induced drought tolerance in cucumber plants at least in part by stimulating the leaf SOD activity to effectively scavenge over-produced O_2_^−^, which could result in increased H_2_O_2_ levels in BBS-treated cucumber plants under drought stress. It is also known that over-produced H_2_O_2_ can be subsequently reduced into H_2_O by POD, CAT, APX, GR, monodehydroascorbate reductase (MDHAR), and dehydroascorbate reductase (DHAR) at different cellular locales [44]–[46]. In the current study, besides stimulating the activity of SOD, BBS also maintained *cAPX* transcription levels (Figure 3A, B), but did not enhance the activities of POD (Figure 4B) and CAT (Figure 4C) in the leaves of cucumber plants under drought conditions. On the other side, the expression of *MDHAR* was undetected in both the BBS and control treatments (data not shown). These results suggested that the mechanisms by which BBS elicited drought tolerance in cucumber plants involved SOD and cAPX; but not MDHAR, POD, or CAT.

It is known that drought stress stimulates ethylene production and emission in plants, resulting in reduced root and shoot growth [47]. On the other hand, ACC deaminase-containing bacteria have been shown to degrade the ethylene precursor ACC via the action of ACC deaminase to release plant stress and rescue normal plant growth, thus enhancing plant drought tolerance [22], [27]. In the present study ACC deaminase activity was undetected in none of the culture solution of three BBS constituent strains (Figure 5). Besides, homologous genes of ACC deaminase were undetected in AR156, SM21 and XY21 genome through PCR (data not showed), which suggested that BBS-elicited drought tolerance in cucumber plants was not due to decreasing plant ethylene levels.

**Figure 6:**
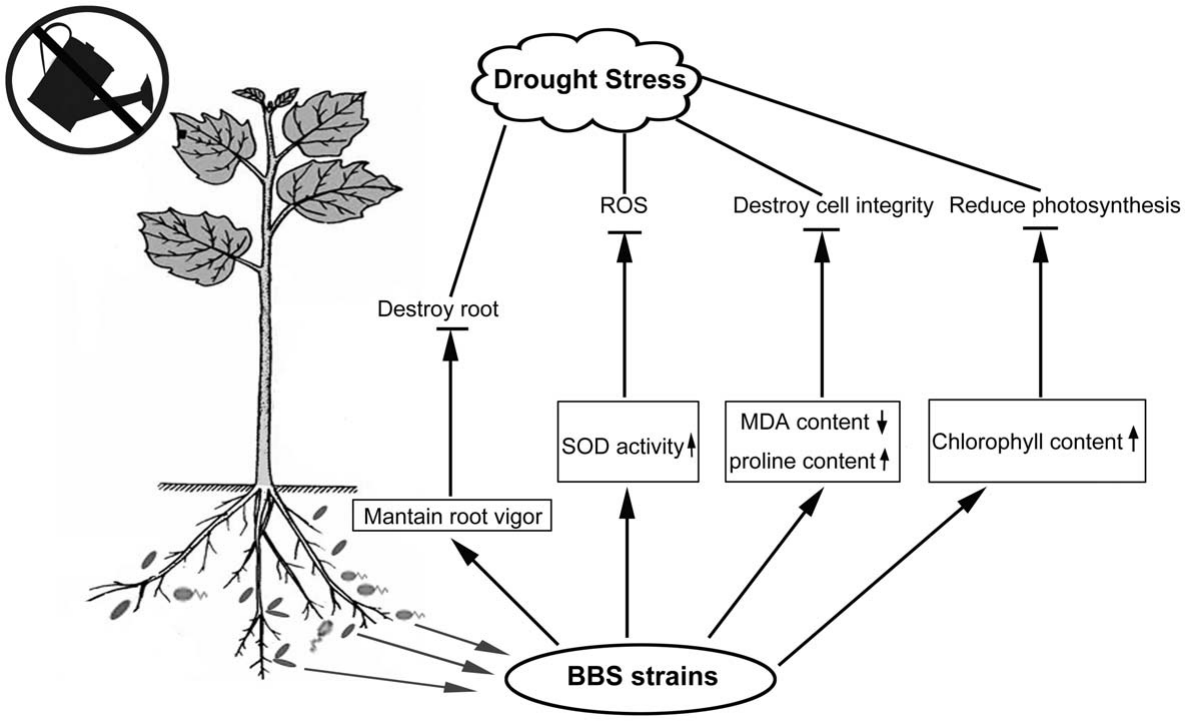
BBS consortium induced systemic tolerance to drought stress in cucumber plants. BBS strains colonized the cucumber roots, maintained a high level of root vigor to protect the root from destroy. Solid arrows indicate plant compounds affected by BBS components. The signal mobilized from the roots to the leaves, which kept SOD activity, proline content, chlorophyll content at higher levels, and kept MDA content at a lower level, to protect the integrity of plant cells, and to release oxidative stress and to remain photosynthesis, leading induced drought tolerance.

Thus, we propose a model of BBS consortium induced drought tolerance in cucumber plants based on the results here (Figure 6). BBS strains colonized cucumber roots, leading to a distinct signal generated in the roots, which maintained a high level of root vigor. The signal mobilizeed from the roots to the leaves, which kept SOD activity, proline content, chlorophyll content at higher levels, and kept MDA content at a lower level, to protect the integrity of plant cells, and to release oxidative stress and to remain photosynthesis, leading induced drought tolerance. However, ACC deaminase was undetected in the drought tolerance induced by BBS, so we need to investigate whether BBS confers IST to drought stress through producing cytokinin and antioxidants, which are known to be involved in the drought tolerance elicited by *Paenibacillus polymyxa* and *Pseudomonas mendocina Palleroni* in the common bean (*Phaseolus vulgaris* L.) and *Lactuca sativa* L. cv. Tafalla [24], [28] in the future to further unravel the mechanism of drought tolerance induced by BBS.

Single strains of PGPR as well as its combination with either *Rhizobium tropici* or mycorrhizal fungi have been reported to act as drought tolerance elicitors [21], [24], [27], [28], but this study is the first to report that a mixture of three PGPR strains can confer tolerance to drought stress in cucumber plants. Our future study is warranted to identify the BBS constituent strain that plays a key role in eliciting IST to drought stress in cucumber and how the three constituent strains (AR156, SM21, and XY21) interact in the IST-elicitation process.

Originally isolated from the rhizosphere soil of tomato plants in our lab [25], AR156 has been demonstrated to induce the resistance against a broad spectrum of pathogens, such as *Pseudomonas syringae* pv. *tomato* DC3000 (DC3000) [25] and *Meloidogyne incognita* Kofoid et White in *A. thaliana* and some other vegetables [48], [49]; and to trigger induced systemic resistance (ISR) to DC3000 in *Arabidopsis* ecotype Col-0 plants by simultaneously activating the salicylic acid (SA)- and jasmonic acid (JA)/ethylene (ET)-signaling pathways in an NPR1-dependent manner [25]. In addition, SM21 and XY21 were both previously isolated from the forest soil in Zhenjiang City of Jiangsu Province, China. The former has been found to achieve the efficacy of 90.18% in controlling bacteria wilt of tomato in the greenhouse (unpublished data); and the later has been shown to effectively colonize plant roots, to accomplish the efficacy of 70% in controlling bacterial wilt of tomato caused by *Ralstonia solanacearum* in the greenhouse and the field [50], and to increase tomato yield by 180–237% [51]. In the future it is also worthy to investigate whether BBS elicits drought tolerance and disease resistance by some common mechanisms.

In conclusion, BBS, a mixture of three PGPR strains, has been shown to confer IST to drought stress in cucumber plants by maintaining the root recovery intension as well as reducing the peroxidation extent of plasma lemma, stabilizing the osmotic potential, maintaining the photosynthesis efficiency, and increasing activities of the antioxidases SOD and cAPX in the leaves; without involving the action of ACC deaminase to lower plant ethylene levels. Therefore, in addition to functioning as an effective biocontrol agent, BBS also has real potential for facilitating plant growth in arid environments as a biotic drought tolerance elicitor.

## Materials and Methods

### Bacterial strains, plants, and drought stress treatments

Bacterial cells of the PGPR strains *Bacillus cereus* AR156, *Bacillus subtilis* SM21, and *Serratia* sp. XY21 were separately grown in Luria-Berta (LB) medium (10 g L^−1^ tryptone, 5 g L^−1^ yeast extract, 10 g L^−1^ NaCl; pH 7.0–7.2) at 28°C with vigorous shaking at 280 r/min for 24 h. Subsequently, bacterial cells were pelleted by centrifugation, washed once with and resuspended in a sterile 0.85% NaCl solution, and adjusted to 5×10^8^ CFU mL^−1^ for use. BBS was prepared by mixing the three bacterial cell suspensions in a ratio of 1∶1∶1 (vol/vol/vol). Besides, *Burkholderia* sp. 5BS21 was isolated from the rhizosphere soil of banana in Zhuhai City of Guangdong Province, China, by our team.

Cucumber (*Cucumis sativus* L. ‘Jinyou No. 1’) seeds were sown in plastic trays filled with the soil of northeast phaeozem of China, rich of humus, which was bought from Jilin province, China; after germination cucumber seedlings were grown in a growth chamber. Fifteen days after sowing, seedlings were transplanted into plastic pots (355.46 cm^3^ of volume) in one seedling per pot; the seedling in each pot was irrigated every other day with 30 mL of water to maintain an optimal moisture level. Cucumber plants were grown in a greenhouse maintained at day/night temperature of 25°C/18°C with 600 µmol photons m^−2^ s^−1^ of light supplied for 12 h during the daytime. The soil used in the research was all sterilized in 121°C for 1 h, and it repeated three times.

Fifteen days after transplantation seedlings were subjected to the BBS and control treatments in 24 seedlings per treatment. In the BBS treatment 20 mL of BBS cell suspension at 5×10^8^ CFU mL^−1^ was poured on the soil around the roots of the seedling in each pot, and then watering was withheld. Each control seedling was treated as described above except for replacing the BBS cell suspension with an equal volume of sterile water. In a preliminary experiment of drought stress treatment, all cucumber plants showed moderate drought stress symptoms (cucumber leaves no longer rehydrated at night or in an early morning) after withholding watering for 13 days. Therefore, specific physiological indicators of drought tolerance in BBS-treated and control cucumber plants were measured 13 days post inoculation (dpi) as described below.

### Determination of MDA content, relative electrical conductivity, proline content, root vigor, and chlorophyll content

The leaf MDA content was determined at 13 dpi according to the method described by Qiu et al. [7]. Three leaf tissue samples were collected from each treatment; each sample of 0.3 g fresh weight (FW) was homogenized in 5 mL of 5% trichloroacetic acid (TCA), and the homogenate was then centrifuged for 15 min at 8000× g. One milliliter of the resultant supernatant of each sample was mixed with 2.5 mL of thiobarbituric acid (TBA), and the mixture was heated at 100°C in a water bath for 20 min and then immediately cooled on ice. The mixture was subsequently centrifuged at 10000×g for 5 min, and the absorbance of the resulting supernatant was measured at 532 nm and 600 nm. By subtracting the non-specific absorbance at 600 nm, the MDA content in cucumber leaves was determined by its molar extinction coefficient (155 mM^−1^ cm^−1^) and expressed as µmol MDA g^−1^ FW.

The relative electrical conductivity in cucumber leaves was measured at 13 dpi using the method of Yang et al. [52] with minor modifications. First, three leaf samples were collected from each treatment; and then each sample of 0.1 g FW was minced, placed into a cuvette, and mixed with 10 mL of distilled water. After the mixture was incubated in a calorstat set at 32°C for 2 h, its initial electronic conductivity (S1) was measured. Next, the mixture was boiled at 100°C for 30 min and then cooled to room temperature (25°C) to determine its final electric conductivity (S2). Distilled water was used as the blank control, whose electronic conductivity (S0) was measured. The relative electric conductivity (REC) was evaluated using the formula: REC = (S1−S0)/(S2−S0)×100.

The free proline content in cucumber leaves was determined at 13 dpi according to the method of Dobrá et al. [53] with minor modifications. Three leaf samples were collected from each treatment, and each sample of 0.2 g FW was homogenized in 10 mL of 3% aqueous sulfosalicylic acid. After the homogenate was filtered through a piece of filter paper, 1 mL of filtrate was mixed with 1 mL of acid-ninhydrin reagent and 1 mL of glacial acetic acid in a test tube, and then the mixture was heated at 100°C for 1 h. The reaction was terminated by placing the test tube in an ice bath. Subsequently, 5 mL of toluene was added into the test tube, which was then vigorously shaken by hand for 10–15 s and finally incubated at room temperature for 20 min. The absorbance of the upper layer of the mixture containing toluene was measured at 520 nm, with toluene as a blank control.

Root vigor is a reliable and sensitive indicator to evaluate drought tolerance, and it reflects water and nutrition absorbing abilities of drought stressed plants. It was measured according to the triphenyltetrazolium chloride (TTC) method and showed as root recovery intension [54]. Three samples of white young roots were collected from each treatment. Each sample of 0.5 g FW was placed into a test tube and mixed with 5 mL of 0.4% TTC and 5 mL of phosphate buffer (0.06 mol L^−1^, pH 7.0). After the mixture was incubated at 37°C for 3 h, the chemical reaction was terminated by adding 2 mL of 1 mol L^−1^ sulfuric acid into the tube. The roots were subsequently transferred to a mortal containing 3–4 mL of ethyl acetate and a little quartz sand, and ground with a pestle. The liquid phase was transferred to a test tube, to which ethyl acetate was added to bring the total volume of the mixture to 10 mL, and then its OD values were measured at 485 nm with a UV-vis recording spectrophotometer (UV1000 Spectrophotometer) for calculating equivalent triphenyl formazan (TTF) concentrations according to the TTC standard curve generated in this experiment. The root activity was determined for per gram (FW) of a root sample using the following formula:
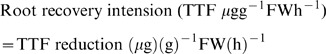



The contents of leaf chlorophylls *a*, *b*, and *a*+*b* were determined at 13 dpi using the method of Ashraf et al. [55]. Three leaf samples were collected from each treatment, and each sample of 0.2 g FW was cut into 0.5 cm segments and extracted overnight with 80% acetone at −10°C. The mixture was centrifuged at 14000× g for 5 min, and the absorbance of the resulting supernatant was measured at 645 and 663 nm using a spectrophotometer (Hitachi-220). The contents of leaf chlorophylls *a*, *b* and *a+b* were calculated according to the following formulas:
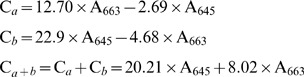


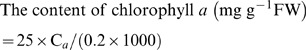


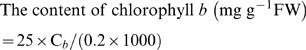


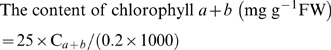



### Enzyme assays

The activities of SOD, POD, and CAT in cucumber leaves were assayed according to the method of Qiu et al. [7]. Three leaf samples were collected from each treatment at 0, 5, 7, 9, 11, and 13 dpi; and each sample of 0.2 g FW was placed into a mortar with 2 mL of 50 mM ice-cold phosphate buffer (pH 7.8) containing 1 mM ethylene diamine tetraacetic acid (EDTA) and homogenized with a pestle. The homogenate was centrifuged at 15000× g for 15 min at 4°C. The supernatant was an enzyme extract containing SOD, POD, and CAT, which was used in the following enzyme assays performed at 4°C.

The leaf SOD activity was determined on the basis of its effectiveness in inhibiting the photoreduction of nitro blue tetrazolium (NBT). Upon the addition of riboflavin into a test tube containing 3 mL reaction mixture [50 mM phosphate buffer (pH 7.8), 0.1 mM EDTA, 130 mM methionine, 0.75 mM NBT, 0.02 mM riboflavin, and 0.1 mL of the enzyme extract], the tube was illuminated with two 20 W fluorescent lamps, which lasted for 10 min. Non-illuminated and illuminated reactions without the enzyme extract served as calibration standards. The absorbance values of the reaction mixture and the blank control were measured at 560 nm with a UV1000 Spectrophotometer. One unit of SOD activity (U) was defined as the amount of enzyme required to cause 50% inhibition of the NBT photoreduction rate, and the results were expressed as U mg^−1^ of FW. The leaf SOD activity was calculated according to the following formula:
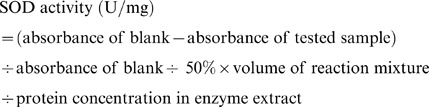



The leaf POD activity was determined based on the oxidation of guaiacol using hydrogen peroxide. The reaction was initiated by adding 20 µL of the enzyme extract to 3 mL of reaction mixture consisting of 100 mM phosphate buffer (pH 7.0), 20 µL of guaiacol solution, and 10 µL of hydrogen peroxide solution. The absorbance was measured at 470 nm at time points of reaction initiation and 5 min later with a UV1000 Spectrophotometer. Enzyme activity was quantified based on the variation of absorbance per minute using the extinction coefficient (26.6 mM^−1^ cm^−1^) according to the following formula:
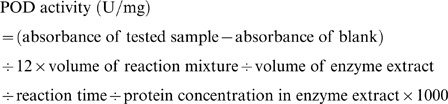



The leaf CAT activity was determined based on the decrease in H_2_O_2_ levels. The reaction mixture (3 mL) consisted of 100 mM phosphate buffer (pH 7.0), 0.1 µM EDTA, 0.1% H_2_O_2_, and 0.1 mL of enzyme extract. The reaction was initiated by adding the enzyme extract to the reaction mixture. The decrease in H_2_O_2_ levels was determined by measuring the absorbance at 240 nm with a UV1000 Spectrophotometer, and quantified by using extinction coefficient (36 M^−1^ cm^−1^). The leaf CAT activity was calculated using the following formula:
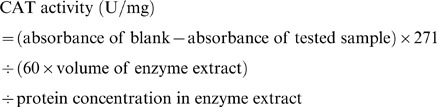



### Analysis of gene expression by RT-PCR

To analyze the expression patterns of the genes *cAPX* (D88649.1), *rbcL* (EF208123.1), and *rbcS* (EF208124.1), cucumber leaf samples were collected at 0, 5, 7, 9, 11, 13 dpi. RT-PCR was performed according to the manufacturer's instructions of PrimeScriptTM 1st Strand cDNA Synthesis Kit (TaKaRa, Japan). Total RNA was extracted from each sample using TRIzol® reagent (Invitrogen, USA). Using the Oligo dT Primer, first-strand cDNAs were synthesized from 1000 ng of total RNA. Independent PCR with 25 cycles was performed using aliquots (1 µL) of cDNA samples. A constitutively expressing gene *Actin* (AB010922.1) was used as a quantitative control in the RT-PCR analysis. Primer Premier 5.00 was employed to design specific primer pairs of *cAPX* (forward primer: TTGTTGCTGTTGAGGTTA, reverse primer: TGAGAGGGTTGGTAGTCC), of *rbcL* (forward primer: ATCTTGGCAGCATTCCGAGTA, reverse primer: CCCAATAGAGGGCGACCAT), and of *rbcS* (forward primer: ACAGGTCACCAGGATACTACGA, reverse primer: CCTCAAGAAAGCCTCAGCA).

Relative mRNA levels of genes were analyzed based on densitometry values obtained using the Quality One software of Bio-Rad.

### Detection of ACC deaminase

The PGPR strains AR156, SM21, XY21, and 5BS21 were examined for the production of ACC deaminase according to the method of Chen et al. [56] with minor modifications as described below. After grown in MSA medium [2 g L^−1^ NaNO_3_, 1.2 g L^−1^ K_2_HPO_4_, 0.5 g L^−1^ MgSO_4_, 0.5 g L^−1^ KCl, 0.14 g L^−1^ KH_2_PO_4_, 0.01 g L^−1^ Fe_2_(SO_4_)_3_·H_2_O, 0.1 g L^−1^ yeast extract paste, 15 g L^−1^ agar; pH 7.2] with ACC (0.5 g L^−1^) (ACC+) and that without ACC (ACC−) at 28°C with shaking at 120 r/min for 48 h, bacterial cells of each strain were centrifuged at 14000× g for 5 min. Five microliters of the supernatant resulting from each culture was spotted on a thin layer chromatography (TLC) silica gel plate, with the same volume of MSA medium with ACC (ACC+) and that without ACC (ACC−) as controls. The TLC plate was subsequently immersed in a developing solution consisting of n-butyl alcohol, acetic acid, and distilled water in the ratio of 15∶1∶2 (vol/vol/vol). After developed for 2.5 h, the silica gel was stained with 0.5% ninhydrin for 20 min in an attemperator set at 100°C. After staining, ACC deaminase-containing bacterium was indicated by the disappearance of khaki on the silica gel plate.

### Statistical analysis

Data of physiological indexes associated with drought tolerance and of antioxidase activities were analyzed statistically at factorial level by means of variance analysis (ANOVA), and their significance levels (p<0.05 or p<0.01) were determined using the statistical software data processing system (DPS version 7.05).
